# Metformin-associated lactic acidosis: risk factors and prognostic factors

**DOI:** 10.1186/cc12391

**Published:** 2013-03-19

**Authors:** V Di Falco, A Milano, M Battilana, F Araosta, A Grosso, D Albanese, F Petrini, L Di Liberato

**Affiliations:** 1 ASL Chieti SS. Annunziata, Chieti, Italy

## Introduction

Metformin, an oral hypoglycemic drug, belongs to the biguanide class and is now generally accepted as first-line treatment in type 2 diabetes mellitus, especially in overweight patients [1]. In some predisposing conditions, the use of metformin may result in metformin-associated lactic acidosis (MALA), a rare adverse event associated with a high mortality rate [2]. The aim of this study is to assess risk factors and prognostic factors in patients with MALA.

## Methods

We conducted a retrospective study of patients with MALA admitted to the ICU of ASL 2 Chieti between 1 January 2008 and 30 September 2012. The eligibility criteria were: diagnosis of diabetes mellitus type 2, treatment with oral hypoglycemic drugs containing metformin, increased anion gap metabolic acidosis (pH <7.35, HCO_3 _<22 mmol/l, lactate >5 mmol/l). For each patient we evaluated: sex, age, home care, SAPS II score [3], blood tests.

## Results

Ten patients were selected, five males and five females, with a mean age of 72.2. On admission, nine of 10 patients had a framework of general illness and acute renal failure; one patient appeared with a probable acute abdomen not subsequently confirmed on CT. Eight patients had a state of dehydration resulting from gastroenteritis, diarrhea, fever; two patients had taken NSAIDs in the days prior to hospitalization. No patient, in these conditions, has discontinued treatment with metformin. All patients showed a critical clinical framework with a SAPS II mean score of 77.5. Cardiovascular and respiratory support was required in all cases. The hemogas analysis showed that patients had a severe metabolic acidosis (mean pH = 6.96) with increased plasma lactate (mean lactate = 15.93 mmol/l). Prothrombin activity was normal in eight of 10 patients. The overall mortality was 70%.

## Conclusion

Patient education on correct use of metformin is essential to prevent MALA, specially in those clinical conditions of increased risk (for example, acute renal failure), as recommended by the AIFA 2011 guidelines [4]. In our study, a higher plasma concentration of lactate represents the main negative prognostic factor, as pointed out by other studies [5]. The prothrombin activity, which is considered to be a decisive prognostic factor in the study of Peters and colleagues [6], was not impaired in patients with poor outcome.

## References

[B1] UK Prospective Diabetes Study Group Lancet 1998 352 854 865 9742977

[B2] Lalau JD Drug Saf 2010 33 727 740 10.2165/11536790-000000000-00000 20701406

[B3] Le Gall JR JAMA 1993 270 2957 2963 10.1001/jama.1993.03510240069035 8254858

[B4] Italian Medicines Agency 2011 http://www.agenziafarmaco.gov.it/it/content/raccomandazioni-sull%E2%80%99utilizzo-dei-medicinali-base-di-metformina-nella-gestione-del-diabete-m

[B5] Dell'Aglio DM Ann Emerg Med 2009 54 818 823 10.1016/j.annemergmed.2009.04.023 19556031

[B6] Peters N Crit Care 2008 12 R149 10.1186/cc7137 19036140 PMC2646313

